# E2F1 enhances glycolysis through suppressing Sirt6 transcription in cancer cells

**DOI:** 10.18632/oncotarget.3594

**Published:** 2015-03-14

**Authors:** Minghui Wu, Edward Seto, Jingsong Zhang

**Affiliations:** ^1^ Department of Genitourinary Oncology and Department of Cancer Imaging and Metabolism, H Lee Moffitt Cancer Center, Tampa, FL, USA; ^2^ Department of Molecular Oncology, H Lee Moffitt Cancer Center, Tampa, FL, USA

**Keywords:** SIRT6, E2F1, glycolysis, metabolism, HDAC1

## Abstract

The fast proliferation of cancer cells requires reprogramming of its energy metabolism with aerobic glycolysis as a major energy source. Sirt6, a class III histone deacetylase, has been shown to down regulate glycolysis by inhibiting the expression of several key glycolytic genes. Based on the published study on the metabolic phenotype of E2F1 −/− mice and SIRT6 −/− mice, we hypothesize that E2F1 enhances glycolysis and inhibits the expression of Sirt6. Indeed, over-expressing of E2F1, but not its DNA binding deficient mutant, significantly enhanced glucose uptake and lactate production in bladder and prostate cancer cell lines. E2F1 over-expression also suppressed Sirt6 expression and function. Moreover, E2F1 directly bound to Sirt6 promoter and suppressed Sirt6 promoter activity under both normoxic and hypoxic culture conditions. E2F1 siRNA blocked the up-regulation of E2F1 under hypoxia, increased Sirt6 expression and decreased glycolysis compared to those of scrambled siRNA transected cells. Furthermore, HDAC1 deacetylated E2F1 and diminished its transcription suppression of Sirt6 promoter. Treatment with the HDAC inhibitor, trichostatin A (TSA), suppressed Sirt6 promoter activity with increased binding of acetylated E2F1 to Sirt6 promoter. Mutating the E2F1 binding site on the proximal Sirt6 promoter abolished the suppression of Sirt6 transcription by TSA. These data indicate a novel oncogenic role of E2F1, i.e. enhancing glycolysis by suppressing Sirt6 transcription.

## INTRODUCTION

As a founding member of E2F family of transcription factors, E2F1 participates in regulating cell-cycle progression, cell differentiation, DNA repair, and apoptosis. Depending on the cellular context and environmental conditions, it can function as either an oncogene or a tumor suppressor gene. Early studies indicated that dysregulation of E2F1 predicted invasive bladder and skin tumors, and also mediated chemoresistance [1]. Further studies with the E2F1 −/− mice revealed enhanced oxidative metabolism and diminished glycolytic metabolism in brown adipose tissue (BAT) and gastrocnemius muscle (GNM) [2]. This phenotype is mediated through enhanced expression of genes involved in oxidative metabolism and diminished expression of glycolytic genes in BAT and GNM of E2F1 −/− mice [2]. In the past few years, accumulating evidence has highlighted the role of SIRT6, a member of the class III histone deacetylases in regulating cell metabolism [3-4]. Through studies of SIRT6 −/− mice and SIRT6 −/− mouse Embryonic Stem cells, Zhong et al showed that SIRT6 co-repressed the hypoxia-inducible factor, HIF-1a, by deacetylating histone 3 lysine 9 (H3K9) in the promoter of key glycolytic genes like Glucose transporter type1 (GLUT1) and Pyruvate Dehydrogenase Kinase, Isoenzyme 1 (PDK1) [3]. SIRT6 −/− mice therefore have uncontrolled glycolysis and died by 1 month of age due to a precipitous decrease in serum glucose. Enhanced aerobic glycolysis was mostly observed in BAT and muscle [3]. This metabolic phenotype is in direct contrast of the E2F1 −/− mice, suggesting that E2F1 may down regulate SIRT6 to facilitate glucose metabolism.

Reprogramming of energy metabolism has emerged as a new hallmark of cancer. Genetic alterations in RAS and MYC have been shown to create addictions of cancer cells to certain metabolic pathways [5]. We hypothesize that the oncogenic role of E2F1 can also be attributed to its promotion of glycolysis through suppressing SIRT6 expression and activity in cancer cells. The only published report on SIRT6 regulation to date came from studies with a mouse hepatoma cell line, Hepa1-6, which showed that a complex of SIRT1, FOXO3a and NRF1 up-regulated SIRT6 upon nutritional stress [6]. It is unclear whether this complex directly regulated SIRT6 transcription at the promoter region. In our study, we find that there is an E2F binding site at the proximal SIRT6 promoter. Although E2F1 has been shown to primarily function as a positive regulator of transcription, it can function as a transcriptional repressor either alone [7-8] or through association with specific co-factors, such as HDAC1 [9] and DNMT1 [10]. Among different cancer types, E2F1 overexpression has been linked to tumor progression in prostate and bladder cancers [11-12], which indicates a tumor promoting role of E2F1 in these cancer types. In addition to inhibiting aerobic glycolysis, SIRT6 has been shown to promote genomic stability through facilitating DNA repair [13-15], attenuate inflammation through damping NF-κB-dependent gene expression [16], and function like a tumor suppressor through repressing Warburg effect in cancer cells [17].

Here we tested our hypothesis in bladder and prostate cancer cell lines and found that E2F1 could indeed suppress SIRT6 transcription and promote cancer cell glycolysis. Mechanistically, E2F1 directly bound to the proximal SIRT6 promoter and formed a complex with HDAC1. Deacetylation of E2F1 by HDAC1 facilitated SIRT6 transcription by dissociating E2F1 from the SIRT6 promoter region.

## RESULTS

### Over-expression of E2F1 enhances glycolysis through repressing SIRT6

**Figure 1:**
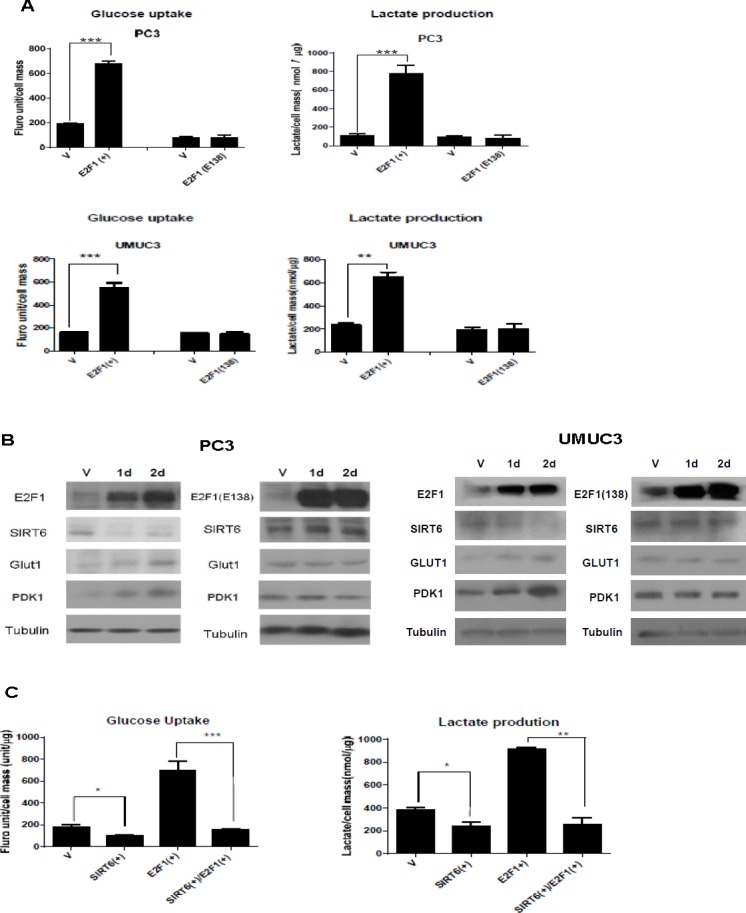
Overexpression of E2F1, not its DNA binding deficient mutant, E2F1(E138), enhances glycolysis through repressing SIRT6 (A) Over-expression of E2F1, but not E2F1(E138), enhanced glucose uptake and lactate production in PC3 and UMUC3 cells. Glucose uptake and lactate production were measured 48 h after transfection. (B) Western blots comparing levels of SIRT6, GLUT1, and PDK1 after over-expression of E2F1 or E2F1 (E138) in PC3 and UMUC3 cells. Tubulin was used as loading control. (C) Over-expression of SIRT6 blocked the enhanced glucose uptake and lactate production induced by E2F1 over-expression in PC3 cell. Three replicates were performed for each experiment. Each column represents the mean and SEM of 3 independent experiments. V: empty vector.*P < 0.05, **P < 0.01, ***P < 0.001.

To test the role of E2F1 in regulating glycolysis, we over-expressed E2F1 or its DNA binding deficient mutant E2F1 (E138) in PC3 prostate cancer and UMUC3 bladder cancer cells. Compared to pcDNA3.1 vector transfected cells, a significant increase in glucose uptake and lactate production was observed after over expressing E2F1, but not E2F1 (E138) (Figure 1A). Compared to vector or E2F1 (E138) transfected cells, E2F1 over-expression led to decreased SIRT6 and increased expression of key glycolysis proteins, GLUT1 and PDK1. Both GLUT1 and PDK1 transcriptions were normally repressed by SIRT6 [3]. The lack of activity with over-expressing E2F1 (E138) indicates that E2F1 needs to bind to DNA to enhance glycolysis (Figure 1B).

To test further that SIRT6 is involved in the enhanced glycolysis after E2F1 over-expression, glucose uptake and lactate production were compared among pcDNA3.1 vector transfected PC3 cells and PC3 cells over-expressing SIRT6, E2F1, or both. As shown in Figure 1C, glycolysis was suppressed after SIRT6 over-expression and enhanced after E2F1 over-expression. Over-expression of both E2F1 and SIRT6 had a glycolysis level similar to SIRT6 over-expression. These data indicate repressing SIRT6 is required in the regulation of glycolysis by E2F1.

### E2F1 binds to SIRT6 promoter and suppresses its transcription

**Figure 2:**
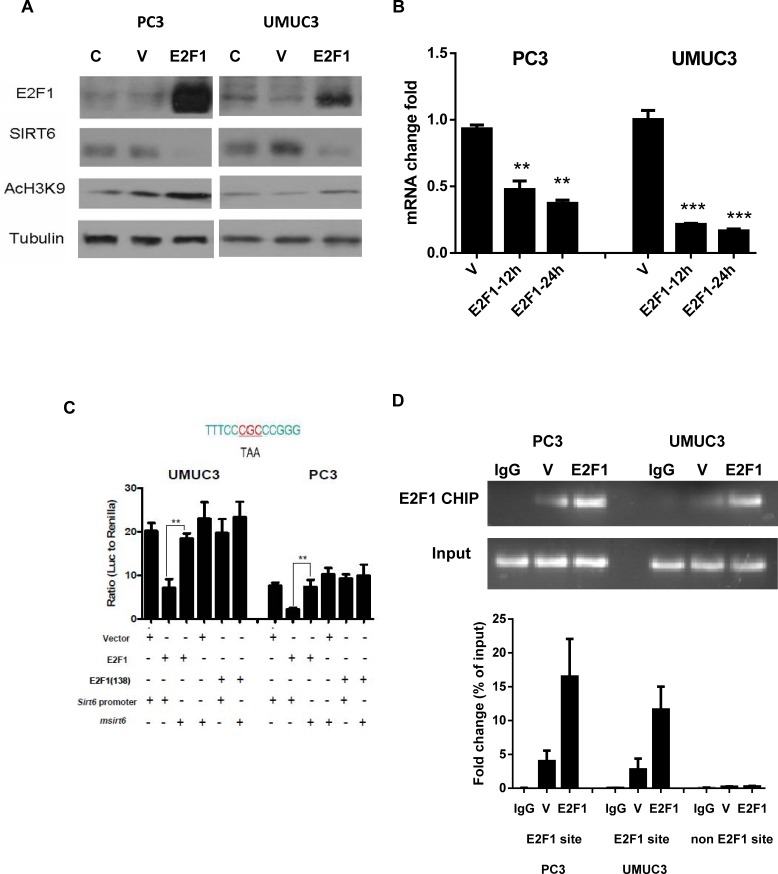
Over-expression of E2F1 reduces SIRT6 transcription through direct binding to *SIRT6* promoter (A) E2F1 over-expression reduced SIRT6 protein and its histone deacetylase activity. (B) Reduction of *SIRT6* mRNA levels at 12 hours and 24 hours post transfection as detected by qRT-PCR in pcDNA-E2F1 transfected cells (E2F1) versus vector transfected cells (V). (C) Dual reporter luciferase assay comparing SIRT6 promoter activities with different combinations of pcDNA3 constructs [vector, E2F1, E2F1(138)] and SIRT6 promoter PGL3 reporter constructs (SIRT6 and SIRT6 with mutated E2F1 binding site, mSIRT6). The sequence of the E2F1 binding site on the proximal SIRT6 promoter and mutated sites on this sequence were shown above (D) Compared to vector transfected cells (V), E2F1 over-expression (E2F1) enhanced binding of E2F1 to the SIRT6 promoter on the ChIP assays. Representative gel pictures were shown above and the intensities of E2F1 binding were quantitated as bar graphs below. Each bar represents the mean and SEM of 3 independent experiments. *P < 0.05, **P < 0.01, ***P < 0.001.

As a class III histone deacetylase, SIRT6 is known to deacetylate the acetylation at H3K9 [3]. Indeed, decreased SIRT6 protein after over-expressing E2F1 in PC3 and UMUC3 cells led to increased H3K9 acetylation compared to vector transfected cells (Figure 2A). The decrease in SIRT6 after E2F1 over expression was also detected at the RNA level (Figure 2B). Of note, there is an E2F1 binding site at the proximal SIRT6 promoter. No potential E2F1 binding sties were identified on the GLUT1 or PDK1 promoter. We therefore performed dual reporter luciferase assay after cotransfecting the pcDNA-E2F1 construct with the SIRT6 promoter pGL3 reporter construct. Compared to pcDNA3.1 vector transfected cells, over-expressing E2F1 suppressed SIRT6 promoter activity. Minimum suppressions of Sirt6 promoter activities were observed after cotransfecting the DNA binding deficient E2F1(E138) with the SIRT6 promoter reporter construct (Figure 2C). We then mutated the E2F1 binding site on the Sirt6 promoter with strategies reported previously [18]. Neither E2F1 nor E2F1 (E138) could suppress Sirt6 promoter activities when the Sirt6 promoter with mutated E2F1 binding site was used in the reporter assay (Figure 2C).

To confirm that E2F1 directly binds to SIRT6 promoter, ChIP assay was performed on SIRT promoter with anti-E2F1 pull down. Compared to empty vector transfected cells, over expression of E2F1 led to increased binding of E2F1 to the SIRT6 promoter as detected by the ChIP assay (Figure 2D). A 200 bp fragment, 1kb upstream of the SIRT6 promoter E2F1 binding site, was amplified in the lysate pulled down with anti-E2F1. Given there is no known E2F1 binding site in this region, the absence of this fragment served as a negative control.

### Up- regulation of E2F1 and its suppression of SIRT6 transcription under hypoxia

**Figure 3:**
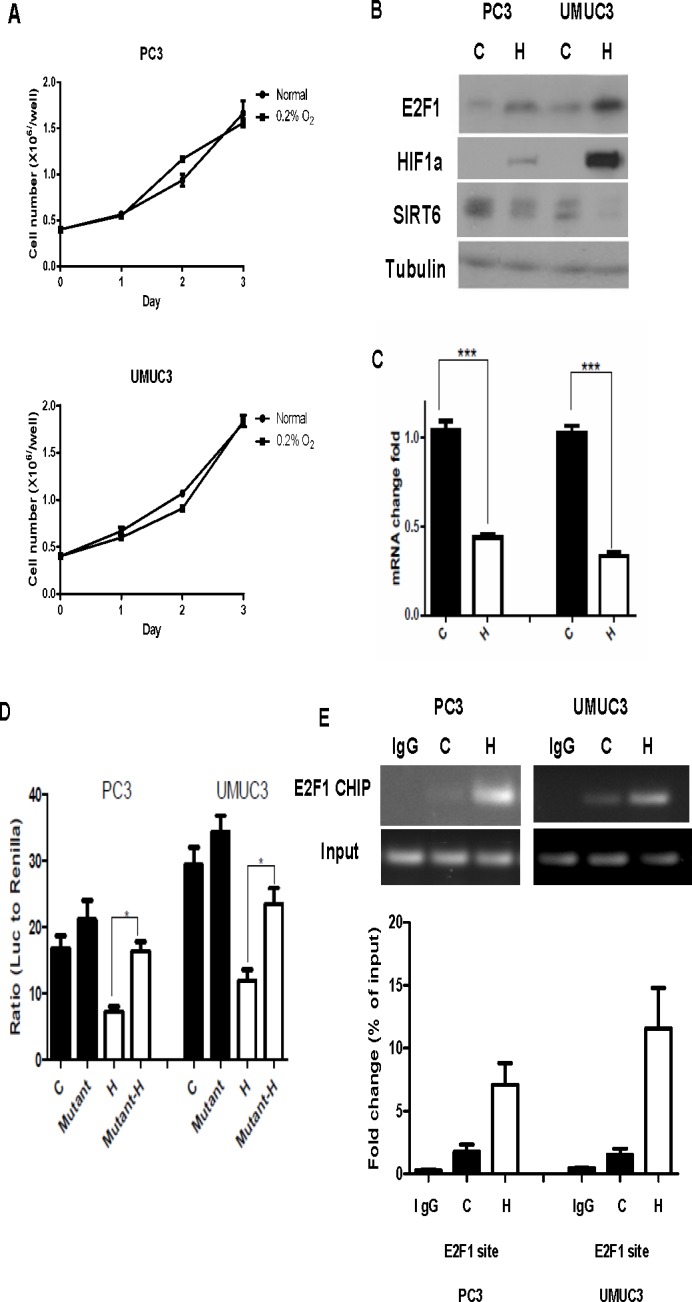
Increased E2F1 and its binding to SIRT6 promoter under hypoxia (A) Growth curve of PC3 and UMUC3 cells showed continued proliferation under hypoxia (0.2% O2) compared to normoxia. (B) Western blot comparing levels of E2F1, HIF1a and SIRT6 in PC3 and UMUC3 cells grew under normoxia (C) and hypoxia (H). (C) Compared to normoxia (C), SIRT6 mRNA level was significantly decreased after 24 hours of hypoxic exposure (H). (D) Dual reporter luciferase assay comparing promoter activities of wild type and mutated E2F1 binding site under normoxia (black bars) and hypoxia (white bars). (E) ChIP assays demonstrated increased E2F1 binding to SIRT6 promoter region in PC3 and UMUC3 cells following 12 hours of hypoxic exposure compared to normoxia. Three replicates were performed for each experiment. Each column represents the mean and SEM of 3 independent experiments.*P < 0.05, **P < 0.01, ***P < 0.001.

Glycolysis has been shown to be enhanced under hypoxia through down-regulation of SIRT6 and up-regulation of glycolytic genes regulated by HIF-1α [3]. It is unclear how SIRT6 is down- regulated under hypoxic conditions. We next studied the E2F1 and Sirt6 interaction under a physiological condition, like hypoxia. Both prostate and bladder cancer cell lines with mutant p53 continue to proliferate under chronic hypoxia (Figure 3A). Compared to cells under normoxic culture conditions, increase in E2F1 and HIF-1α and decrease in SIRT6 were observed 24 hours after exposure to 0.2% of oxygen (Figure 3B). The decrease in SIRT6 under hypoxia was also detected at the RNA level (Figure 3C).

SIRT6 promoter activities with or without the mutation at the E2F1 binding site were then compared between normoxia and hypoxia with dual reporter luciferase assays. Compared to those under normoxic conditions, SIRT6 promoter activity was significantly decreased in PC3 and UMUC3 cells under hypoxia. The recovery of SIRT6 promoter activity after mutating E2F1 binding site on its proximal promoter was seen under normoxia as well as hypoxia, but significant at a greater extent under hypoxia (Figure 3D). Consistent with what we observed with the E2F1 over-expression experiment (Figure 2D), the increase of E2F1 under hypoxia was associated with increased binding of E2F1 to the SIRT6 promoter in the CHIP assay (Figure 3E).

### Knocking down E2F1 increases SIRT6 expression and function

**Figure 4:**
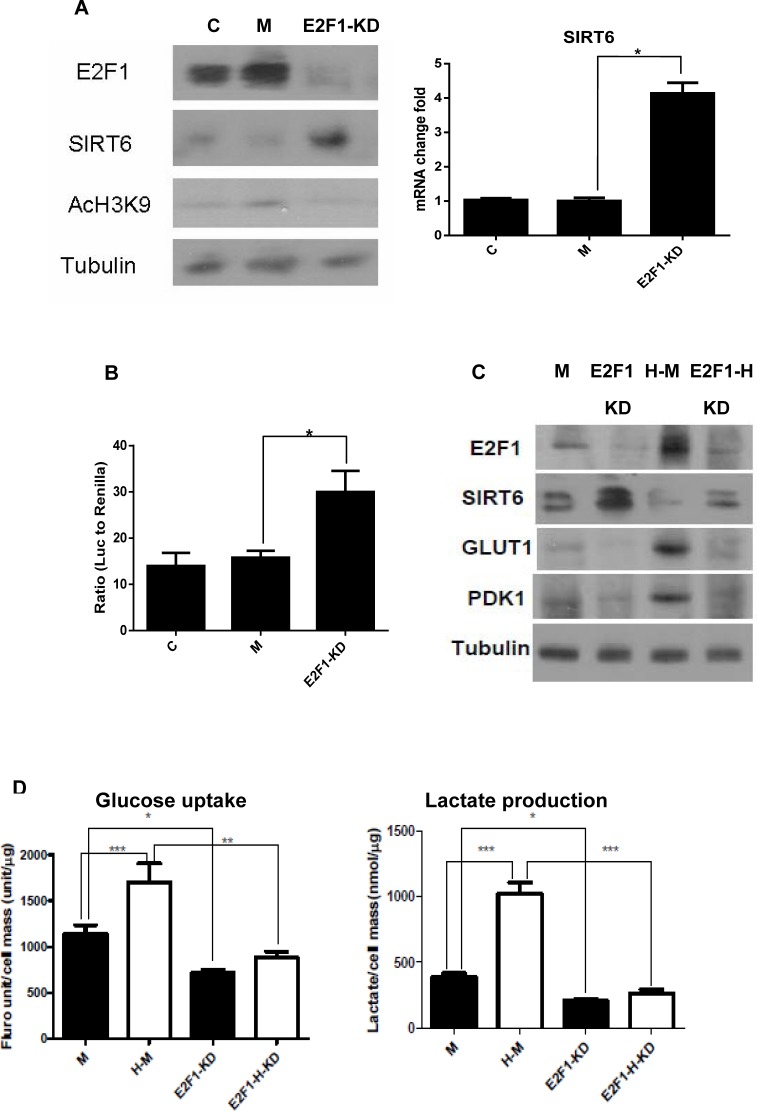
Knocking down E2F1 increases SIRT6 and decreases glycolysis (A) E2F1 silence increased SIRT6 protein and its histone deacetylase activity in UMUC3 cells (*left*). Increase of *SIRT6* mRNA was detected by qRT-PCR in E2F1 siRNA transfected cells (E2F1-KD) compared to untransfected (C) and control siRNA transfected cells (M) (*right*). (B) Compared to untransfected (C) and control siRNA transfected cells (M), dual reporter luciferase assay showed E2F1 silence (E2F1-KD) increased SIRT6 promoter activity. (C) Compared to control siRNA transfected cells (M), E2F1 knock down (KD) increased SIRT6 and decreased GLUT1 and PDK1 under normoxic and hypoxic (H) conditions in UMUC3 cell. (D) Glucose uptake and lactate production were inhibited after E2F1 knock down under normoxic and hypoxic conditions in UMUC3 cells. M: mock siRNA; H-M: mock siRNA under hypoxia; E2F1-KD: E2F1 knock-down; E2F1-H-KD: E2F1 knock-down under hypoxia. Three replicates were performed for each experiment. Each column represents the mean and SEM of 3 independent experiments. *P < 0.05, **P < 0.01, ***P < 0.001.

To test further the negative regulation of SIRT6 by E2F1, siRNA was used to inhibit E2F1 expression (Figure 4A). Compared to un-transfected and scrambled siRNA transfected UMUC3 cells, knocking down E2F1 with siRNA increased SIRT6 protein, RNA and promoter activity (Figure 4A and 4B). The increases in Srit6 and decreases in GLUT1 and PDK1 proteins after knocking down E2F1 were observed under both normoxia and hypoxia (Figure 4D). Consistent with these changes at the gene expression level, significant decreases of glucose uptake and lactate production were observed at the function level after knocking down E2F1 with siRNA under both normoxic and hypoxic culture conditions (Figure 4E). Given glycolysis was enhanced under hypoxia; the decrease in glycolysis after knocking down E2F1 was more significant under hypoxia compared to normoxia (Figure 4E). These data support further that increased E2F1 expression and subsequent down-regulation of SIRT6 expression is a main mechanism for enhanced glycolysis in these cell lines.

### HDAC1 deacetylates E2F1 and diminishes its negative regulation of SIRT6 promoter

**Figure 5:**
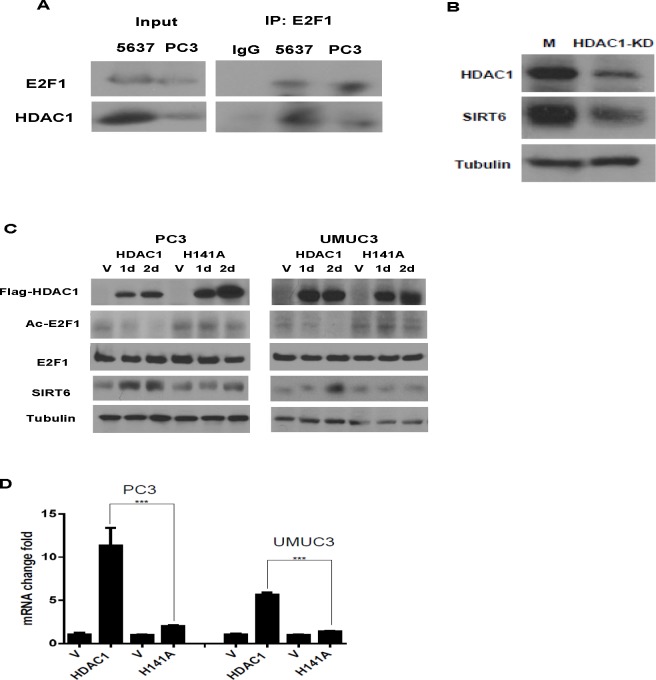
HDAC1 forms a complex with E2F1 and up regulated SIRT6 expression (A) HDAC1 was immuno-precipitated by anti-E2F1 antibody in PC3 and 5637 cells. (B) HDAC1 knock down (KD) deceased SIRT6 protein in PC3 cell compared to control siRNA transfected cells (M). (C) Western blots of HDAC1, Ac-E2F1, E2F1 and SIRT6 after over-expression of HDAC1 or its mutant (H141A) in PC3 and UMUC3 cells. The HDAC1(H141A) mutant has no deacetylase activity. (D) Over expressing HDAC1, not HDAC1(H141A), increased SIRT6 mRNA level. Three replicates were performed for each experiment. Each column represents the mean and SEM of 3 independent experiments. V: empty vector; M: mock siRNA. *P < 0.05, **P < 0.01, ***P < 0.001.

HDAC1 has been shown to be recruited to the promoters of E2F1 target genes. It deacetylates E2F1 and diminishes the binding of E2F1 to its downstream gene promoters [19]. We then studied whether HDAC1 used the same mechanism to diminish the regulation of SIRT6 transcription by E2F1. As shown in Figure 5A, complex formation between E2F1 and HDAC1 was detected with immunoprecipitation in bladder and prostate cancer cells (Figure 5A). Knocking down HDAC1 with siRNA decreased SIRT6 level in PC3 cell (Figure 5B). To study whether the deacetylase activity of HDAC1 is involved in this regulation, H141A, an HDAC1 mutant that lacks the histone deacetylase activity, or the empty vector was then over-expressed in PC3 and UMUC3 cells. Compared to their empty vector transfected counterparts, decreases in E2F1 acetylation and increase in SIRT6 RNA and protein were detected after over-expressing HDAC1, but not HDAC1 (H141A) (Figure 5C and 5D).

**Figure 6:**
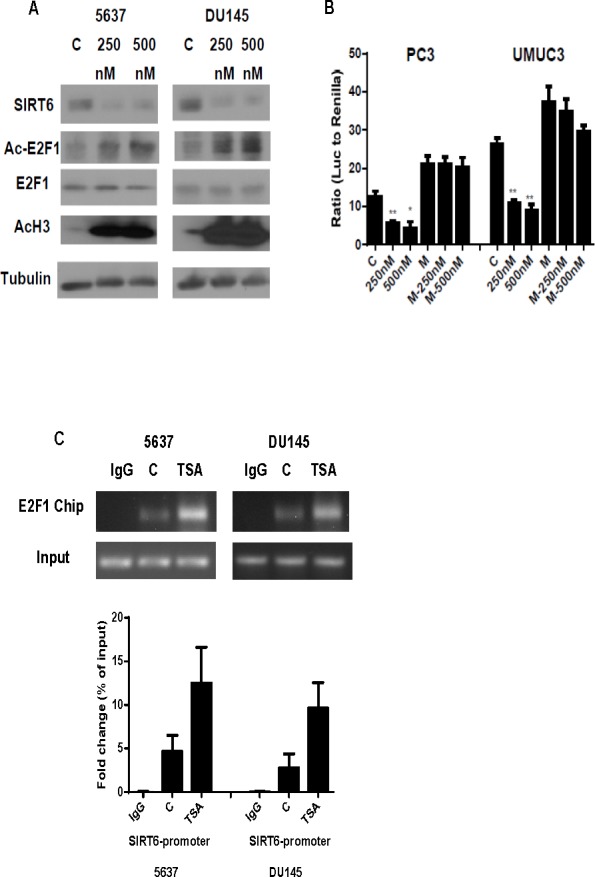
HDAC inhibitor enhances E2F1 acetylation and its binding and suppression of SIRT6 promoter (A) Western blot comparing levels of SIRT6, Acetylated (Ac) E2F1, E2F1 and Acetylated (Ac) H3 with or without TSA treatment in DU145 and 5637 cells after 250 and 500nM TSA treatment. (B) Dual luciferase reporter assay showed significantly decreased SIRT6 promoter activity following TSA treatment (250 and 500 nM) in wild type, but not E2F1 binding site mutated SIRT6. (C) ChIP assays showed increased E2F1 binding to SIRT6 promoter region in DU145 and 5637 cells after 500nM TSA treatment compared to untreated control (C). Three replicates were performed for each experiment. Each column represents the mean and SEM of 3 independent experiments. V: empty vector; M: E2F1-binding mutation. *P < 0.05, **P < 0.01, ***P < 0.001.

To test further that deacetylation of E2F1 by HDAC1 increases SIRT6 transcription, 5637 and DU145 cells were treated with HDAC inhibitor, TSA. As shown in Figure 6A, a dose dependent increase in acetylation of E2F1 and histone 3 was acetylation were detected in 5637 and DU145 cells. This is associated with a dose-dependent decrease in SIRT6 expression and SIRT6 promoter activities (Figure 6A and 6B). TSA treatment, however, had minimal effects on SIRT6 promoter activities when the E2F1 binding site on the proximal SIRT6 promoter was mutated. To test whether increased E2F1 acetylation after TSA treatment enhances its binding to the SIRT6 promoter, Chip assay was performed with anti E2F1 pull down. Compared to the E2F1 levels on the SIRT6 promoter of untreated cells, higher levels of E2F1 on the SIRT6 promoter were detected after TSA treatment (Figure 6C). These data support further that HDAC1 deacetylates E2F1 and diminishes its negative regulation of SIRT6 promoter.

## DISCUSSION

Utilizing prostate cancer and bladder cancer cell lines, our data support our hypothesis that E2F1 suppresses SIRT6 transcription and facilities cancer cell glycolysis. Unlike its well documented roles in regulating cell-cycle progression, cell differentiation, DNA repair, and apoptosis, the role of E2F1 in promoting cancer cell aerobic glycolysis has not been reported before. Studies on BAT and GNM of E2F1 −/− mice have reported that E2F1 inhibits oxidative metabolism and enhances glycolytic metabolism [2]. E2F1 has also been shown to up-regulate pyruvate dehydrogenase kinase 4 (PDK4) transcription in C2C12 myoblasts and human cardiac AC16 cells [20-21]. Like PDK4, PDK1 inhibits pyruvate dehydrogenase activity, and facilities lactate production by diverting pyruvate away from the tricarboxylic acid (TCA) cycle. Based on the published data, PDK1 seems to play a bigger role in regulating this process compared to its other isoforms, particularly in cancer cells [17]. Our data showed that E2F1 up-regulates the expression and function of key glycolysis regulators like PDK1 and GLUT1 through repressing the transcription of SIRT6, a master regulator of glycolysis [3]. Although our data do not exclude the possibility that E2F1 may directly regulate PDK1 and or GLUT1, no standard E2F1 binding sites were identified in the PDK1 and GLUT1 promoter regions. Furthermore forced over-expression of SIRT6 can reverse the glucose uptake and lactate production induced by E2F1 over-expression (Figure 1C), which indicates that repressing SIRT6 is required in the regulation of glycolysis by E2F1. Given several key proteins in the glycolysis pathway are negatively regulated either directly or indirectly by SIRT6; repressing SIRT6 transcription is a more effective way for E2F1 to up regulate aerobic glycolysis compare to directly activating individual glycolytic genes.

Bladder cancer and prostate cancer cells are chosen for this study based on the oncogenic role of E2F1 reported in these 2 malignancies [11-12]. In the case of bladder cancer, an E2F1 gene expression signature has been associated with muscle invasive bladder cancer and been shown to predict progression of superficial bladder cancers to the more aggressive muscle invasive bladder cancers [12]. High E2F1 expression is associated with high levels of GLUT1 and PDK1 in these bladder and prostate cancer datasets [11, 12]. Given detecting under expressed genes is not as robust as detecting over expressed gene with gene expression array; we recently used immunohistochemistry to study SIRT6 expression in muscle invasive bladder cancers obtained through radical cystectomy [22]. Consistent with the down-regulation of SIRT6 by E2F1, The expression of SRIT6 was significantly diminished when bladder cancers invaded deeper, i.e. progressed further on the tumor staging. Functional studies with bladder cancer cell lines indicate that a major function of SIRT6 in bladder cancer is to down-regulate aerobic glycolysis [22]. A highly glycolytic phenotype is also recently reported in a metastatic prostate cancer mouse model established with luciferase expressing PC3 cells [23]. These published data based on *in vivo* models and primary tumor samples support our *in vitro* finding that E2F1 down regulates SIRT6 transcription and facilitates aerobic glycolysis.

Despite SIRT6's important roles in regulating glycolysis, DNA repair and genome integrity, the upstream regulation of SIRT6 expression and function has not been well studied. A significant increase in E2F1 and decrease in SIRT6 was observed within 24 hours of hypoxic culture (Figure 3B). The decrease in SIRT6 expression and function under hypoxia was significantly diminished after knocking down E2F1 with siRNA (Figure 4D). More importantly, our data showed that HDAC1 deacetylates E2F1 and diminished its negative regulation of SIRT6 promoter and increased SIRT6 mRNA and protein expressions. Based on the reported tumor suppressor role of SIRT6, the decreased SIRT6 expression after treatment with HDAC inhibitor, TSA, reflects the complexity of developing HDAC inhibitor for cancer treatments (Figure 6). Our finding on the repression of SIRT6 transcription by E2F1 will broaden the therapeutic opportunities to enhance SIRT6 tumor suppressor activities with compounds like CDK4/6 inhibitor, which retains E2F1 in the RB-E2F1 complex by diminishing the phosphorylation of RB by CDK4/6.

## EXPERIMENTAL PROCEDURES

### Cell culture

Prostate cancer (PC3 and DU145) and bladder cancer (5637 and UMUC3) cell lines were obtained from American Type Culture Collection (ATCC) and maintained in culture mediums as instructed by ATCC, supplemented with 10% FBS and 1% penicillin. For hypoxia experiments, cells were incubated in a hypoxic chamber (Biospherix) with constant 0.2% oxygen.

### Western blot

Protein lysate preparation and immunoblotting were performed as described previously [18]. Rabbit anti-SIRT6 polyclonal antibody (Cell Signaling), rabbit anti-PDK1 polyclonal antibody (Cell Signaling), rabbit anti-E2F1 polyclonal antibodies (Cell Signaling), mouse anti-Glut1 monoclonal antibody (Abcam), rabbit anti-HDAC1 polyclonal antibody (Santa Cruz), mouse anti-flag monoclonal antibody (Sigma), rabbit anti-AcH3K9 (Cell Signaling) polyclonal antibody and rabbit anti-AcH3 (Millipore) were used as the primary antibodies. Tubulin was used as the loading control (Sigma). Immunoreactive protein was detected using ECL reagents (Roche) according to the manufacturer's instructions.

### Immunoprecipitation

Cells were grown to about 70% confluency, then washed twice with 1xPBS, and collected in CHAPS buffer (25 mM HEPES, 2 mM EGTA, 2.5 mM MgCl2 and 0.3% CHAPS). After 20 min of incubation on ice and 10 min of centrifugation at 4°C, 500 μg of Lysates were pre-cleared with 30 μl Protein A/G Agarose beads (Invitrogen) for 2h and supernatants were collected. IPs were performed by mixing supernatants with 3 μg of E2F1 (Santa Cruz) or HDAC1 (Santa Cruz) antibodies or IgG (Abcam), together with 30 μl of protein A/G Agarose beads, for overnight at 4°C. After 3 washes of PBS buffer, the immuno-complexes were eluted by 10 min boiling in 2x SDS loading buffer.

### Real time RT-PCR assay

Total RNA was extracted using RNeasy Mini kit (Qiagen); 2 μg total RNA was reversely transcribed using SuperScript II Reverse Transcriptase (Applied Biosystems) according to the manufacturer's instructions. Sequences of SIRT6 primers were as follows: 5”-CTTGGCACATTCCACAA-3” (forward) and 5”-GCTTCCTGGTCAGCCAGA-3” (reverse). Amplification reaction assays contained 1× SYBR green PCR Mastermix (Applied Biosystem) at the optimal concentrations, and amplification was performed using an ABI PRISM 7000 SDS thermal cycler (Applied Biosystem). GADPH was used as the reference gene for normalization.

### Quantitative Chromatin Immunoprecipitation (ChIP) Analysis

ChIP experiments were performed following the protocol of Chip-IT Express Kit (Active Motif). Briefly, cells were cross-linked with formaldehyde and incubated with 1X lysis buffer with a protease-inhibitor mixture, and sonicated to generate 200-500bp DNA fragments. After incubation with 5 μg of anti-E2F1 antibody (Santa Cruz) or anti-HDAC1 antibody (Santa Cruz) and cross-linking reversal, bound DNA was obtained by phenol chloroform extraction and ethanol precipitation, and resuspended in 50μl of H2O. PCR was performed with 5 μl of immunoprecipitated target DNA. Input material corresponding to 1% of total sample was recovered prior to inmunoprecipitation, and PCR was performed with 1 μl of purified DNA. Primer sets used were the following: 5′-GGCTCTGTCCTTACGGAATTTA −3′(forward) and 5′ TCTTTGCATGCA GGTGTTTG −3′ (reverse). The final immunoprecipitation (IP) products were monitored by real-time PCR. Input signal was treated as 100% binding. Relative promoter occupancy was calculated by comparing the cycle numbers between input (representing 1% of total lysate) and IP samples.

### Transfection of siRNA

SIRT6 siRNA pools were purchased from Santa Cruz. E2F1 and HDAC1 siRNA Smartpools (4 target specific siRNAs) were purchased from Dharmacon. Cells were seeded in 6-well plates and transfected with 5μM siRNAs. After 6 hours, the medium with siRNA was removed and cells were incubated with fresh medium 1-2 days before further treatment. A mock siRNA (Dharmacon) was used as control.

### Transfection of plasmid

cDNA 3.1 Plasmid encoding E2F1 and its mutant (E138) were kindly provided by Dr. Doug Cress. Cells were seeded in 6-well plates and transfected with 2 μg plasmids. After 6 hours, the medium with plasmid was removed and cells were incubated with fresh medium for two days. An empty vector (invitrogen) was used as control.

### Luciferase reporter gene assays

Promoter region of SIRT6 about 1.4kb was cloned into PGL3 luciferase reporter vector. Cells were seeded in 96-well culture plates (Costar) and transfected with 2 ng/well Renilla luciferase construct along with 0.2 μg/well of each SIRT6 promoter firefly luciferase reporter construct in triplicate. Firefly and Renilla luciferase activities were measured with the Dual-Luciferase Reporter Assay kit (Promega).

### Site-directed mutagenesis

Mutation on *SIRT6* E2F1 binding consensus was generated by site-directed mutagenesis using the QuikChange Kit from Stratagene according to previous publication [18] and the pGL3 clones as template. E2F binding consensus and mutated sequence on *SIRT6* promoter region are in Figure 2C. The introduction of mutations was confirmed by sequencing.

### Glucose and lactate uptake assays

For glucose uptake assays, cells were grown under normoxic or hypoxic condition for 24 hours and 150 μM 2-NBDG (Invitrogen) was added to the media. Fluorescence was measured in a FACSCalibur Analyzer (BD). Lactate concentration was measured in the media by using the Lactate Assay Kit (Biovision).

### Statistical analysis

Statistical analysis was performed using the GraphPad Prism 5 software. For one-way ANOVA, Tukey's multiple comparison test was used. T-test was used for two-group comparisons. Data derived from at least 3 independent experiments were shown as means ± SEM. *P < 0.05, **P < 0.01, ***P < 0.001.
